# Gene Co‐Expression Networks Highlight Key Nodes Associated With Ammonium Nitrate in Sugarcane

**DOI:** 10.1111/ppl.70612

**Published:** 2025-10-31

**Authors:** Jorge Mario Muñoz‐Pérez, Denis Bassi, Lucia Mattiello, Marcelo Menossi, Diego Mauricio Riaño‐Pachón

**Affiliations:** ^1^ Laboratório de Biologia Computacional, Evolutiva e de Sistemas, Divisão de Produtividade Agroindustrial e Alimentos, Centro de Energia Nuclear na Agricultura Universidade de São Paulo Piracicaba São Paulo Brazil; ^2^ Bayer SA ‐ Centro de Desenvolvimento de Produtos Petrolina Pernambuco Brazil; ^3^ Instituto de Biologia, Departamento de Genética, Evolução, Microbiologia e Imunologia Universidade Estadual de Campinas Campinas São Paulo Brazil; ^4^ Centro de Melhoramento Molecular de Plantas Universidade Estadual de Campinas Campinas São Paulo Brazil

**Keywords:** gene co‐expression network, MYB transcription factors, nitrogen use efficiency (NUE), sugarcane, transcriptomics

## Abstract

This study aimed to investigate molecular mechanisms underlying nitrogen use efficiency (NUE) in sugarcane by analyzing transcriptome profiles of two genotypes (responsive, RB975375; non‐responsive, RB937570) under contrasting nitrogen conditions, and along the leaf development gradient. Using RNA‐seq data from 48 leaf segment samples, the analyses integrated gene co‐expression network approaches to uncover genotype‐specific regulatory modules and metabolic pathways linked to NUE. The responsive genotype prioritized carbon metabolism and defense under high nitrogen, while the non‐responsive genotype activated photosynthesis and stress responses. Co‐expression analysis revealed 44 nitrogen‐responsive and 20 genotype‐correlated modules. Module 20, enriched in MYB/MYB‐related transcription factors, emerged as a central regulator of nitrogen response. Key metabolic markers (RUBISCO, PEPCASE) correlated with specific modules, and novel candidate genes (e.g., NewTr2475430.gen) showed genotype‐specific expression. Generated resources include (1) RNA‐seq datasets (NCBI BioProject PRJNA1176579); (2) a *de novo* transcriptome assembly (3.8 million transcripts clustered into 2.48 million transcript groups); (3) co‐expression networks (1109 nodes, 199 modules); (4) annotated DEGs (2723) and metabolic correlations (e.g., RUBISCO, chlorophyll); (5) genotype‐specific expression profiles and candidate genes (e.g., MYB transcription factors, uncharacterized transcripts). This resource provides actionable targets (e.g., MYB TFs, uncharacterized transcripts) for improving NUE in sugarcane breeding. The network modules and metabolic correlations offer a systems‐level framework to study complex nitrogen‐responsive mechanisms in polyploid C4 crops. Publicly available datasets enable comparative studies on nutrient use efficiency in polyploid crops, advancing sustainable agriculture.

## 1 Introduction

Sugarcane is one of the most efficient crops in converting solar energy into sugars, capable of accumulating up to 0.7 M sucrose in mature stalks (Moore 1995). Despite this impressive efficiency, the theoretical maximum could be 70%–80% higher under optimal conditions (Waclawovsky et al. 2010). Achieving this potential requires a deeper understanding of sugarcane physiology, particularly its photosynthetic performance. Unlike wheat and rice, where high photosynthetic rates have been a breeding focus (Makino 2011), sugarcane breeding has traditionally prioritized traits such as sugar content, nutritional demands and stress resistance (Lu et al. 2024; Zhang et al. 2025), even though enhanced photosynthesis could significantly boost productivity and biomass (Long et al. 2006; Marchiori et al. 2014).

Sugarcane has a C4 photosynthetic metabolism, which is highly regulated by the source/sink balance (Wardlaw 1990; McCormick et al. 2008). This metabolic pathway, coupled with environmental factors like CO_2_ concentration, light, temperature, nutrient and water availability, plays a crucial role in the plant's photosynthetic activity (Meinzer and Zhu 1998; Koonjah et al. 2006; Vu et al. 2006; De Souza et al. 2008; Yamori et al. 2014). Thus, enhancing crop photosynthesis is inherently complex, spanning processes from light capture and electron transport to carbon fixation and nitrogen allocation.

In this context, nitrogen use efficiency (NUE), and specifically photosynthetic NUE (PNUE) is pivotal, as it has the capacity to constrain photosynthetic capacity and its plastic responses to stress (Congreves et al. 2021; Govindasamy et al. 2023). Nitrogen (N) promotes protein synthesis, cellular growth, and the expansion of photosynthetic area, which in turn affects the production and translocation of photo‐assimilates (Ryle et al. 1979; Theobald et al. 1998; Lawlor 2002; Vos et al. 2005; Kraiser et al. 2011; Leghari et al. 2016). In sugarcane, photosynthesis is closely linked to leaf N content, with a significant portion of N being mobilized from leaves to culms during the vegetative phase, negatively impacting the photosynthesis rate (Allison et al. 1997; Meinzer and Zhu 1998; Park et al. 2005). N supply also modulates the establishment of photosynthesis along the developing leaf, and shapes primary metabolism, underscoring a tight C–N integration (Bassi et al. 2018). However, the molecular mechanisms linking leaf development, N, and photosynthesis in sugarcane remain underexplored.

This study represents the first comprehensive application of co‐expression network analysis to investigate how varying N levels applied to two genotypes with contrasting NUE affect the molecular mechanisms underlying leaf development and PNUE in sugarcane.

We hypothesize that N availability modulates PNUE in sugarcane through two complementary mechanisms. On the one hand, as we have shown previously, strong spatial dynamics along the leaf development gradient, particularly the middle and tip regions, define zones of maximal photosynthetic activity (Mattiello et al. 2015; Bassi et al. 2018). On the other hand, findings of co‐expression network analyses suggest that genotype‐specific regulatory rewiring plays an important role. For example, in a recent study, genes involved in N assimilation, photosynthesis and carbon metabolism, originating from the wild sugarcane progenitor *Saccharum spontaneum* , were upregulated in a high‐NUE cultivar, indicating that distinct regulatory networks can drive pathways associated with NUE differences (Hui et al. 2025). Similar findings, genotype‐dependent rewiring of the transcriptional networks in response to N availability, have been shown for rice, maize and sorghum (Zamboni et al. 2014; Zhang et al. 2019; Singh et al. 2022; Liu et al. 2024). By integrating these two perspectives, spatially dynamics and genotype‐dependent network organization, this study aims to identify whether PNUE improvements arise primarily from developmental zonation along the leaf blade or from transcriptional network architecture, and to identify candidate genes and modules central to N‐mediated photosynthetic control in sugarcane.

We identified 2723 groups of differentially expressed genes (DEGs) across various leaf segments and N conditions, revealing clear contrasts between N‐responsive and non‐responsive cultivars. The responsive genotype exhibited coordinated activation of pathways related to carbon metabolism and fine‐tuning photosynthetic light capture, while the non‐responsive genotype displayed uncoupled transcriptional adjustments. Co‐expression network analysis identified modules and candidate genes associated with photosynthesis, gene regulation, ion transport, nitrogen/carbon metabolism and defense/stress responses, representing promising targets for breeding or biotechnology approaches. Together, these results support the hypothesis that N responsiveness in sugarcane is mediated by dynamic transcriptional reprogramming of photosynthesis and metabolism along the leaf development gradient, with N‐responsive genotypes displaying tightly regulated networks that enhance PNUE, while non‐responsive genotypes fail to mount such coordinated responses.

## 2 Materials and Methods

### 2.1 Plant Material

The experimental design was the same as Bassi et al. (2018), but the RNA‐seq data presented here are novel. We describe briefly the experimental setup in the following, but we refer the reader to Bassi et al. (2018) for further details. Contrasting sugarcane genotypes, RB975375 (responsive, R) and RB937570 (nonresponsive, NR), were selected based on NUE screening, involving a pool of 20 genotypes exposed to three N concentrations (10, 90, and 270 mg of N per kg of sand) using ammonium nitrate as the N source as described by Robinson et al. (2008). The culms were sprouted in greenhouse trays filled with vermiculite. After 3 weeks, the plantlets were transferred to plastic pots containing 3.4 kg of washed sand and maintained in a greenhouse at a constant temperature of approximately 28°C. The N was applied at two concentrations: low N (10 mg N kg^−1^ sand) and high N (270 mg N kg^−1^ sand). Applications occurred three times at 15‐day intervals. The experimental design was completely randomized, with three biological replicates per genotype and treatment combination. Each biological replicate consisted of a single plant, and samples were collected from four distinct leaf segments per plant. This resulted in a total of 48 samples. Leaf segments were harvested from leaf +1 (the first fully expanded, photosynthetically active leaf) of three‐month‐old plants, 1 month after the final N application. The leaf blade was divided into four developmental zones: Base Zero (B0): The first 2 cm of the leaf base, representing immature tissue. Base (B): middle region of the first third, transitioning from cell division to elongation. Middle (M): middle region of the second third, with mature photosynthetic cells. Tip (P): final third of the leaf. Segments were collected between 10 a.m. and 2 p.m. to minimize diurnal variation, immediately frozen in liquid nitrogen, and stored for downstream analyses.

### 2.2 RNA Extraction, Library Construction, and Sequencing

Total RNA was extracted from three independent replicates for each sample and treatment using Trizol (Invitrogen), with an additional sodium acetate/ethanol precipitation step to enhance purity. RNA quality and concentration were assessed using gel electrophoresis, a NanoDrop spectrophotometer (Thermo Fisher Scientific), and a Bioanalyzer (Agilent Technologies). Only RNA samples with a minimum RNA Integrity Number (RIN) of 7 were used for library construction. A total of 48 libraries were prepared using the TruSeq Stranded mRNA Sample Prep Kit (Illumina), which enriches poly‐A‐containing transcripts and retains strand information. Clusters were generated on a c‐Bot (Illumina), and paired‐end sequencing was performed on a Hi‐Seq 2500 platform (Illumina) with the TruSeq SBS Kit v3—HS (Illumina). Sequencing was conducted at the LACTAD Facility (University of Campinas, Campinas, Brazil). Raw reads can be found under NCBI bioproject PRJNA1176579.

### 2.3 RNA‐Seq Data Pre‐Processing and Assembly

Short‐reads were pre‐processed using FastQC (Andrews 2010), multiQC (Ewels et al. 2016), BBDuk2 v35.85 (Bushnell 2014) and Trimmomatic v0.38 (Bolger et al. 2014) to remove low‐quality regions, remaining adaptor sequences, chloroplast and mitochondria reads, rRNAs, and rat/mouse contamination. All the reads from the same genotype were used to generate a *de novo* transcript assembly with Trinity 2.14.0 (Grabherr et al. 2011) in strand‐specific mode and setting the kmer value to 25 bp and minimum transcript length of 201. The resulting genotype‐specific transcript assemblies were joined into a comprehensive transcriptome.

### 2.4 Transcript Quantification and Grouping

Transcript abundance was quantified with Salmon v1.10.2 (Patro et al. 2017) using the assembled transcriptome as reference. Due to the high ploidy and alternative splicing isoforms present in sugarcane, some of the assembled transcripts could be quite similar and thus share many sequencing fragments, so we decided to cluster them based on the equivalence classes inferred by Salmon v1.10.2 (Patro et al. 2017) using Terminus (Sarkar et al. 2020). The quantification at the transcript level generated by Salmon was summarized at the level of groups inferred by Terminus, and we will refer to these groups as “transcript groups” hereafter.

### 2.5 Functional Annotation of the De Novo Transcriptome

Predicted peptides using TransDecoder v5.5.0 (Hass 2018) were annotated with the Trinotate pipeline v3.3.2 (Bryant et al. 2017), which includes sequence similarity searches against the Swissprot v2020_06 database with BLASTX and BLASTP v2.8.1 (Altschul et al. 1990), prediction of signal peptides using SignalP v5.0b (Teufel et al. 2022), prediction of transmembrane regions using TMHMM v2.0c (Krogh et al. 2001), identification of ribosomal genes with RNAmmer v1.2 (Lagesen et al. 2007), gene ontology (GO) (Ashburner et al. 2000; The Gene Ontology Consortium et al. 2023), transcription associated proteins (TAPs) domains were identified using Hmmer v3.3.2 (Eddy 2011) against Pfam v34 (El‐Gebali et al. 2019). Protein domains were classified into TAPs families following the rules used in PlnTFDB (Riaño‐Pachón et al. 2007; Pérez‐Rodríguez et al. 2010).

### 2.6 Quantification, Quality Control and Differential Gene Expression Analyses

Quantification by Salmon v1.10.2 (Patro et al. 2017) was imported into R using the tximport package (Soneson et al. 2016). We transformed the raw counts using the *vst* function from DESeq2 (Love et al. 2014), which allows us to stabilize the variance across the range of the mean, handle zero counts (Anders and Huber 2010) and provides a more suitable basis for heatmap representation and co‐expression analyses. We conducted PCA analysis using the *ploPCA* function from DESeq2, which uses the top 500 transcript groups with the highest row variance to assess the grouping of the samples according to the experimental design. To evaluate the effect of N levels on gene expression, we compared the expression profiles in the B0 (Base zero), B (base), M (middle), and P (tip) regions of the leaf from sugarcane plants grown under 10 and 270 mg N kg^−1^ sand. Using the raw counts matrix with normalizing factors computed by DESeq2, we identified differentially expressed transcript groups in eight *N* availability contrasting conditions (Table 1). We used DESeq2 (Love et al. 2014) with parameters *altHypothesis = greaterAbs* and *lfcThreshold = 1* in order to get differentially expressed transcript groups two times above/under the background expression level. *p*‐values were adjusted with the Benjamini‐Hochberg (FDR < 0.05) correction for multiple testing (Benjamini and Hochberg 1995)

**TABLE 1:** Contrasts for differential expression analysis. Nitrogen responsiveness = NR: non‐responsive genotype (RB937570), R: responsive genotype (RB975375), leaf segments = B0: Base 0, B: base, M: medium, P: tip, nitrogen level = 10: 10 mg of N per kg of sand, 270: 270 mg of N per kg of sand. We carried out eight statistical contrasts; in each of them genotype and leaf segment remained constant and only nitrogen level varied.

Contrast	Transcript groups tested
NR‐B0‐10 versus NR‐B0‐270	400,756
NR‐B‐10 versus NR‐B‐270	380,525
NR‐M‐10 versus NR‐M‐270	421,290
NR‐P‐10 versus NR‐P‐270	390,561
R‐B0‐10 versus R‐B0‐270	291,408
R‐B‐10 versus R‐B‐270	266,979
R‐M‐10 versus R‐M‐270	278,658
R‐P‐10 versus R‐P‐270	329,470

### 2.7 Inference of Co‐Expression Network and Modules

All transcript groups detected as differentially expressed in at least one of the contrasts were kept for network inference, and counts after the variance stabilizing transformation were used as expression values for network inference, analysis, and heatmap visualization. We filtered out transcript groups with more than half missing entries and zero variance with the function *goodSamplesGenes* from WGCNA (Langfelder and Horvath 2008). Pearson correlation coefficient between each pair of genes was computed with the *fastCor* function from the HiClimR R package (Badr et al. 2015). Only gene pairs with |Pearson *r*| > 0.90 were kept in the network. Clusters, also called modules, of co‐expressed transcript groups were identified with the Markov cluster algorithm MCL (van Dongen 2000) with an inflation value of 1.8. We estimated the Kullback–Leibler divergence between our graph and several random graph models: Erdős–Rényi (Erdos and Rényi 1960), Small‐world (Watts and Strogatz 1998) and scale‐free (Barabási and Albert 1999) as implemented in the StatGraph R package (Takahashi et al. 2012).

### 2.8 Functional Attribution to Gene Sets and Correlation Analysis

In order to identify the most relevant biological functions in gene sets, we carried out overrepresentation tests (Fisher's exact test adjusted *p*‐value < 0.05) of Biological Processes from the Gene Ontology using the TopGO R package (Alexa and Rahnenführer 2025). The gene sets used comprised the differentially expressed transcript groups in each of the contrasts and the co‐expression modules. *p*‐values were adjusted with the Benjamini‐Hochberg correction for multiple testing (Benjamini and Hochberg 1995). To prioritize modules for downstream analysis, we selected modules correlated with genotype, condition, several metabolites and leaf segment. We used the eigengene of each module, which tends to get only one representative expression pattern of a set of transcript groups using the first principal component of their expression matrix (Zhang and Horvath 2005). Using the eigengene for each module, we were able to calculate their correlation with genotype, N availability and several metabolites determined previously for the exact same samples (Bassi et al. 2018). We assessed correlation using the Spearman correlation coefficient (|Spearman ρ| > 0.7), and *p*‐values were adjusted for multiple testing (199 modules and seven variables) using (Bonferroni < 0.05).

## 3 Results and Discussion

### 3.1 Transcript Assembly, Annotation, Quantification and Exploratory Analyses

**FIGURE 1:**
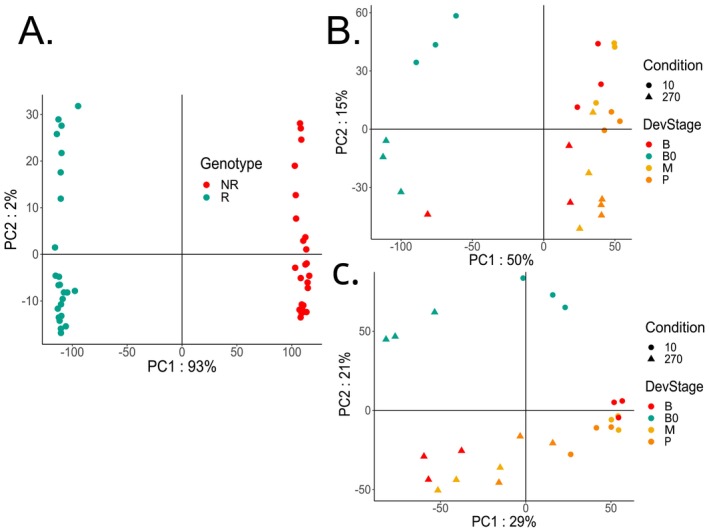
Principal component analysis of variance‐stabilizing transformation (DESeq2) representing the top 500 transcript groups by row variance in each sample. (A) All genotypes. (B) Samples from the *N*‐responsive (R: RB975375) genotype. (C) Samples from the non‐responsive (NR: RB937570) genotype. Leaf segments = B0: Base 0, B: base, M: middle, P: tip, nitrogen level = 10: 10 mg N kg^−1^ sand, 270: 270 mg N kg^−1^ sand.

We assembled 3,835,051 transcripts. Processing with Terminus to exploit equivalence classes resulted in 2,480,471 transcript groups, from which 1,116,882 (45%) encoded at least one protein. 873,612 transcript groups were assigned to 24,063 GO terms, and the 2,502,895 deduced proteins were identified as belonging to 6934 Pfam protein families. Principal component analysis (PCA) of the variance‐stabilized expression data revealed that genotypic differences dominated the expression variance, explaining 93% of total variability, while N treatment and leaf segment accounted for only 2% (Figure 1A). This indicates that the inherent genetic divergence between the two sugarcane genotypes (R and NR) drives the majority of transcriptomic differences, consistent with our previous observations (Bassi et al. 2018). Nonetheless, within genotypes, the samples were grouped according to leaf segment and N condition, confirming that these factors still impart distinct transcriptional profiles (Figure 1B,C).

### 3.2 Different Responses to High N Levels for Sugarcane Genotypes

**FIGURE 2:**
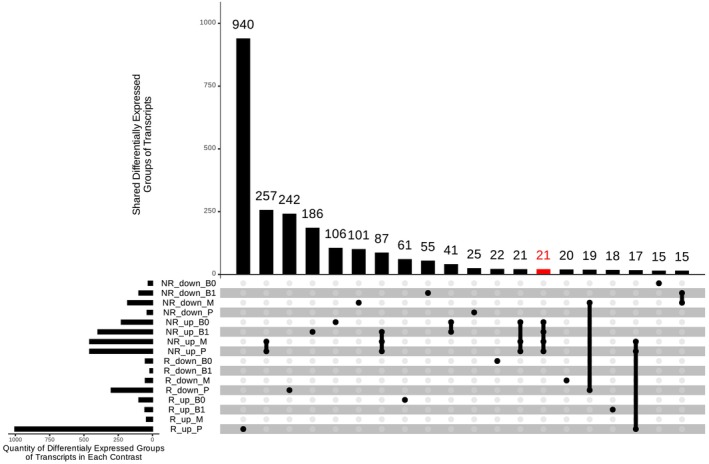
Top 20 sets of differentially expressed transcript groups by size; shared and unique across the different conditions. NR indicates non‐responsive genotype (NR: RB937570), R, responsive genotype (R: RB975375), Up marks the transcript groups overexpressed in high *N* availability (270 mg N kg^−1^ sand). Down point to transcript groups overexpressed in low *N* availability (10 mg N kg^−1^ sand). B0, B1, M and P differentiate Base 0, base, middle and tip leaf segments. Red bar indicates a set of transcript groups always up‐regulated in NR genotype in high N availability irrespective of the leaf segment. A transcript group is considered differentially expressed if |log fold change| > 1 and adjusted *p*‐value < 0.05. *p*‐values were adjusted with the Benjamini–Hochberg (FDR < 0.05) correction for multiple testing. All intersections are available in Figure S1.

Out of the 2,480,471 transcript groups, we identified 2723 differentially expressed (DEGs) across at least one condition. Notably, each genotype exhibited a largely distinct response to high N, with the NR genotype showing a greater number of up‐regulated transcript groups than the R genotype in most leaf segments, except for the tip of the leaf (P) where the R genotype exhibited the greatest number of DEGs compared to all other conditions. Most DEGs were unique for each condition for both genotypes, suggesting different processes occurring in leaf segments. Only a small core of 21 transcript groups in the NR genotype was consistently up‐regulated by high N across all leaf segments (Figure 2). The fact that each leaf segment harbored mostly unique N‐responsive genes highlights the importance of spatial regulation (segment‐specific). Sugarcane leaves develop basipetally, as other grasses do, and prior studies have shown distinct gene expression programs along the leaf gradient (Wang et al. 2014; Mattiello et al. 2015). Our results indicate that N availability modulates these developmental programs, such that each segment mounts a tailored transcriptional response to N that could reflect its developmental needs and metabolic role.

**FIGURE 3:**
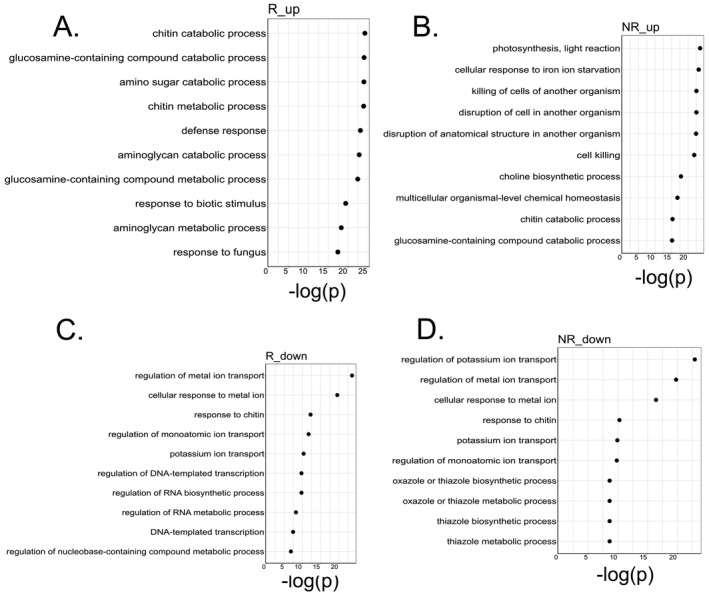
Top 10 GO Biological process functional enrichment of DEGs irrespective of leaf segment. (A) Overrepresented terms in high *N* availability in the responsive (R: RB975375) genotype. (B) Overrepresented terms in high *N* availability in the non‐responsive (NR: RB937570) genotype. (C) Overrepresented terms in low *N* (10 mg N kg^−1^ sand) availability in the R genotype. (D) Overrepresented terms in low *N* availability in NR genotype. Down means overrepresented terms in the low *N* condition (10 mg N kg^−1^ sand), Up refers to the overrepresented terms in the high *N* condition (270 mg N kg^−1^ sand). *p*‐values were calculated using the TopGO R package, and corrected for multiple testing using the Benjamini–Hochberg method, FDR < 0.05.

We carried out GO enrichment in order to understand the biological processes altered by N availability, leaf segment and genotypes. We found that the GO term “response to ammonium ion” GO:0060359 (*p* < 0.01) was overrepresented in the core set of 21 transcript groups consistently up‐regulated by high N in the NR genotype. In the N‐responsive genotype under high N conditions (R_up), the analysis of differentially expressed groups of transcripts across all leaf segments combined revealed that the most relevant overrepresented terms are related to chitin catabolic process, amino sugar catabolic process, and glucosamine‐containing compound catabolic process (Figure 3A). This suggests that the R genotype activates the breakdown of complex sugars, likely reflecting a response to N availability by enhancing carbon metabolism. In contrast, the non‐responsive genotype in high N availability (NR_up) shows photosynthesis (light reaction), redox processes, cellular response to iron ion starvation, cell killing of other organisms and chitin catabolic process as the most relevant terms (Figure 3B). This indicates that the NR genotype emphasizes photosynthetic machinery and stress responses, highlighting a more stress‐oriented reaction rather than a resource‐oriented one. It is also important to note that the enrichment of iron starvation response genes in this genotype, under high N, hints at a possible nutrient imbalance or physiological stress. High N supply can exacerbate iron deficiency in plants, and perhaps the NR genotype is struggling to maintain sufficient iron for chlorophyll synthesis (Sun et al. 2021; Ye et al. 2022; Khalil et al. 2024). In contrast, the R genotype did not show this iron starvation signature under high N, implying a better maintenance of nutrient homeostasis. Instead, R_up uniquely activated many chitinases (47 chitinases‐related transcript groups versus 36 in NR_up). Chitinases are frequently found in responses to biotic and abiotic stress (Vaghela et al. 2022) and N fertilization enhances chitinase levels (Dietrich et al. 2004; Fagard et al. 2014; Maglovski et al. 2017; Sun et al. 2020). For the responsive genotype in low N availability (R_down, i.e., genes upregulated in R under N deprivation), the most relevant terms are regulation of metal ion transport, cellular response to metal ions, and regulation of ion transport (Figure 3C). These data suggest the activation of mechanisms to scavenge and redistribute mineral nutrients, such as iron, zinc, or copper, when N is scarce. The non‐responsive genotype under low N availability (NR_down) similarly overexpressed genes for potassium ion transport and metal ion transport. The prominence of ion transport processes in both genotypes under low N points to a conserved stress adaptation, common with other plants, as nutrient scarcity can disrupt ionic equilibrium and physiological functions (Shao et al. 2020; Ye et al. 2022).

While both genotypes respond to N conditions with shared processes, the responsive genotype (R) prioritizes metabolic and regulatory pathways, especially related to the breakdown of compounds with complex sugars and gene expression regulation. In contrast, the non‐responsive genotype (NR) mounts a more reactive response aimed at bolstering photosynthesis and coping with stress under high N, while under low N, it activates metabolic pathways related to metal ion scavenging and redistribution and the biosynthesis of specialized compounds. This interpretation is supported by physiological data from Bassi et al. (2018). They found that under low N, both genotypes showed stunted growth, but under high N, the R genotype outperformed the NR, exhibiting higher chlorophyll content and delayed leaf senescence, particularly in the upper leaf segments. Our transcriptomic results mirror these findings. For example, in the leaf tip (P segment), which is prone to senescence, the R genotype has the greatest number of N‐responsive genes (Figure 2), many of which are involved in sustaining metabolic processes, whereas the NR genotype did not show such an extensive response. The ability of the R genotype to transcriptionally invest in the leaf tip under high N positively correlated with its higher chlorophyll levels and prolonged functionality of that segment (Bassi et al. 2018). Overall, the differential expression patterns underscore that improving sugarcane NUE is a complex challenge: each genotype employs distinct molecular strategies and different leaf regions mount specialized responses. Breeding for better NUE may thus require combining traits that enhance metabolic flexibility (as in the R genotype) with those that mitigate stress and sustain photosynthesis, addressing the limitations revealed by the NR genotype.

### 3.3 Gene Co‐Expression Network Analysis and Module Identification

To further delve into the coordination of N‐responsive genes, we constructed a gene co‐expression network from the identified DEGs. Out of the set of 2723 DEGs, 1109 with high co‐expression (|Pearson *r*| > 0.90) formed a network with 3699 edges. The network exhibited typical scale‐free properties: it consisted of 110 connected components, with the giant component comprising 56.4% of the nodes. The topology closely matched a Barabási–Albert model of similar size (Kullback–Leibler divergence = 0.08), indicating pronounced clustering and a non‐random organization of co‐expressed nodes within the network. We identified 199 co‐expression clusters (modules) using the Markov clustering algorithm. Module sizes range from two transcript groups up to 73 (6.58% of all nodes in the network); the second largest module had 44 nodes (3.97% of all nodes in the network) and 28 modules (14.07% of all modules in the network) had more than 10 nodes. GO enrichment analyses of each module's members revealed that many modules were significantly overrepresented for specific biological processes (*p*‐value < 0.01), indicating functional coherence within modules. Complete functional GO enrichment for all modules is available under DOI 10.6084/m9.figshare.30100633.

**FIGURE 4:**
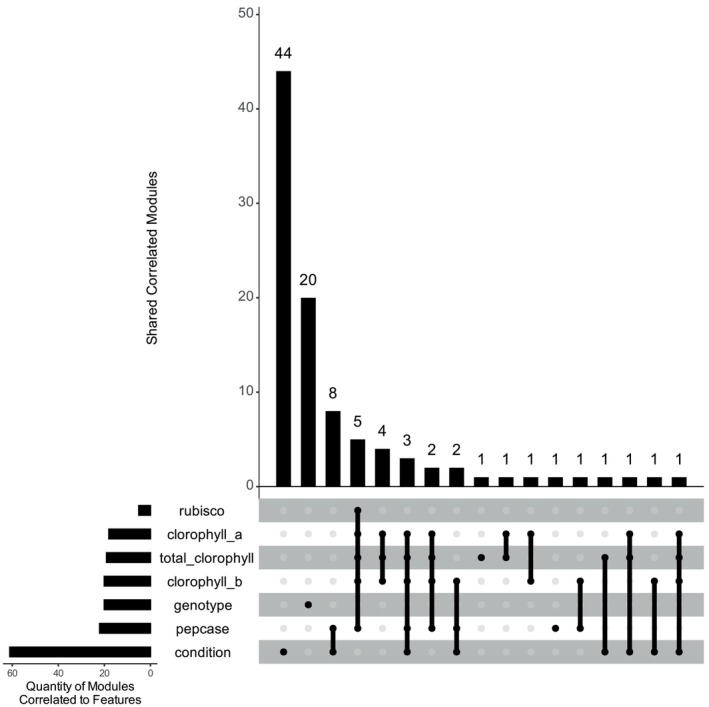
Sets of correlated modules with genotype, *N* condition, total chlorophyll, chlorophyll a, chlorophyll b, rubisco and PEPCase. A module is considered correlated with a feature if the absolute value of the spearman correlation is greater than 0.7, and *p*‐value < 0.05 (Bonferroni correction).

To link these modules with physiological traits and experimental factors, we correlated each module's eigengene (representative expression profile) with genotype, N level, and key leaf traits measured by Bassi et al. (2018), such as total chlorophyll, chlorophyll *a* and *b*, Rubisco, and PEPCase content. This analysis identified 44 modules correlated to N treatment (|Spearman ρ| > 0.7, Bonferroni < 0.05) and 20 correlated to genotype (Figure 4). Notably, a single module expression profile (Module 123, Figure S3 and Table S1) was jointly correlated with both genotype and N level, which underscores the complex, independent genetic architectures underlying NUE and N response. Overall, the segregation of N‐responsive modules versus genotype‐driven modules supports the notion that breeding for improved NUE must address genotype‐specific regulatory networks. This concept aligns with prior agronomic findings that sugarcane genotypes inherently differ in NUE traits, demonstrating genetic variations that can be harnessed in breeding (Robinson et al. 2008). Our network analysis now provides a molecular context for such variation. Given the wealth of modules, we focused on those that were strongly associated with genotype or key physiological traits, reasoning that these modules might contain candidate genes underlying NUE differences. Below, we highlight some of these and their inferred biological roles.

#### 3.3.1 Module 10

**FIGURE 5:**
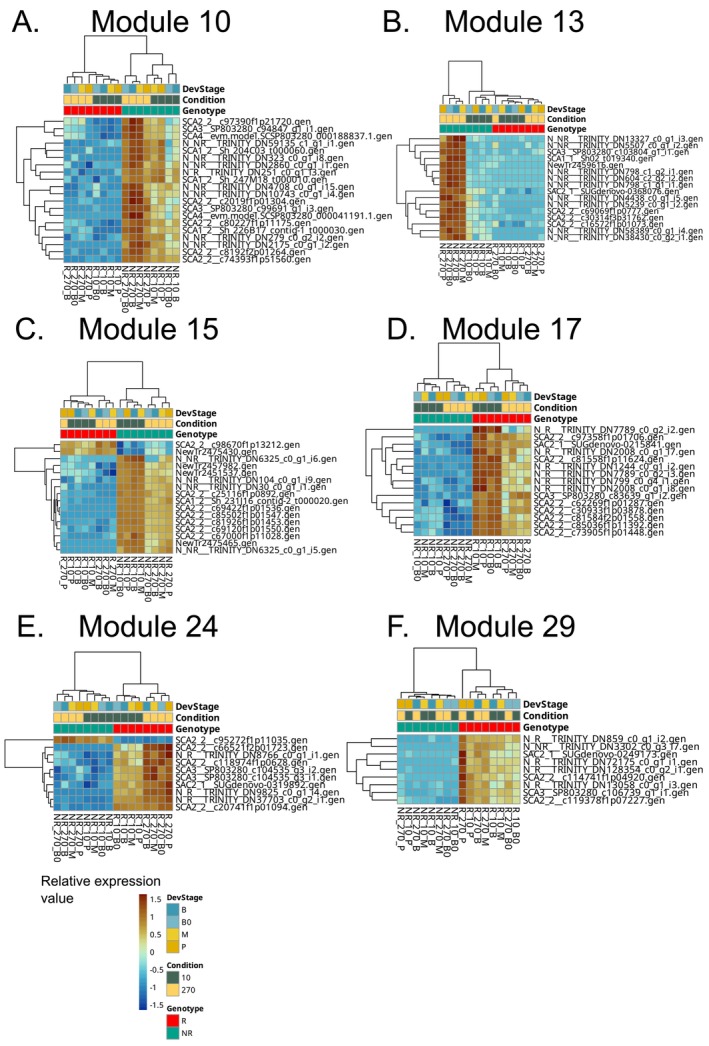
Selected modules responding to genotype. (A) Module 10: enriched in terms involved in cellular energy metabolism, particularly emphasizing photosynthesis, glycolysis, and ATP production. (B) Module 13: enriched in alpha‐amino acid metabolic process, plant organ development, root system development, ammonium ion metabolic process, choline metabolic process. (C) Module 15: enriched in cellular processes related to ion transport, gene expression regulation, biosynthesis of biomolecules, and cellular responses to various stimuli. (D) Module 17: enriched in GO terms suggests involvement in fundamental cellular processes related to responding to stimuli, regulating gene expression, synthesizing macromolecules, and orchestrating developmental processes. (E) Module 24: involved in cellular processes related to protein regulation, amino acid metabolism, and plant defense responses including non‐photochemical quenching and energy quenching. (F) Module 29: enriched in phosphorus and phosphate metabolism, response to external biotic stimulus, response to stress and biotic stimuli, apoptosis, oxylipin biosynthesis and lipid oxidation. Relative expression values correspond to *z*‐score of variance‐stabilized counts from DESeq2. The modules were generated using the Markov Cluster Algorithm (mcl), with an inflation value of 1.8 on the correlation matrix. NR indicates non‐responsive genotype (NR: RB937570), R denotes responsive genotype (R: RB975375). Leaf segments/developmental stage = B0: Base 0, B: base, M: middle, P: tip, nitrogen condition = 10: 10 mg N kg^−1^ sand, 270: 270 mg N kg^−1^ sand.

This module is enriched in transcript groups for core energy metabolism, including photosynthesis, glycolysis, ATP production, monosaccharide metabolism, and oxidation–reduction processes, as well as GO terms related to cytokinin response and organelle organization. The transcript groups in this module have a higher expression level in the NR genotype, especially under high N (Figure 5A). This expression pattern suggests that the NR genotype, when given ample N, channels resources into primary energy generation pathways, likely in an attempt to maximize immediate photosynthetic output and growth, albeit at the cost of longer‐term adjustments. The inclusion of cytokinin response terms could be related to how the NR genotype responds to high fertility by accelerating growth, as cytokinins promote cell division. However, this could lead to imbalances, consistent with NR's signs of stress (e.g., iron starvation), despite enhanced photosynthesis.

#### 3.3.2 Module 13

Enriched in alpha‐amino acid metabolic process, plant organ development, root system development, ammonium ion metabolic process, choline metabolic process, this module was strongly activated only in the NR genotype under high N availability (Figure 5B), where it ramps up amino acid metabolism and perhaps development genes. The enrichment in root development does not necessarily imply root‐specific activity in our leaf samples, but may rather reflect conserved regulatory genes involved in organ development. One interpretation is that the NR genotype, under high N, tries to enhance N assimilation pathways and organ development to uptake even more nutrients. The fact that this module remains largely inactive in the R genotype could point to a less efficient feedback control in NR, i.e., it senses the available N and triggers certain pathways that the R genotype (being NUE‐efficient) does not need to activate as strongly. This divergence points out that even when both genotypes experience the same high‐N, their internal response programs can be quite different.

#### 3.3.3 Module 15

Involved in essential cellular processes related to ion transport, gene expression regulation, biosynthesis of biomolecules, and cellular responses to various stimuli. These processes suggest a role in maintaining cellular homeostasis and adjusting to environmental changes. Remarkably, this module had two transcript groups activated preferentially in the R genotype: SCA2_2__c98670f1p13212.gen and NewTr2475430.gen (Figure 5C). The first one is annotated as coding for phosphoenol pyruvate carboxylase (PEPCase), and it likely contributes to the superior photosynthetic capacity of the R genotype under high N, as it functions as the primary initial CO_2_‐fixing enzyme in mesophyll cells, enabling efficient carbon assimilation and contributing directly to high photosynthetic rates and CO_2_‐use efficiency (Zhu et al. 2010). This is also aligned with the observations in Bassi et al. (2018), in which carbon isotope discrimination measurements evidenced a greater enhancement of PEPCase‐mediated carboxylation when supplied with high N. The second transcript group could not be annotated, making it an intriguing novel candidate for NUE.

#### 3.3.4 Module 17

Enriched GO terms suggest involvement in fundamental cellular processes related to response to stimuli, regulation of gene expression, macromolecule biosynthesis, and developmental processes. Interestingly, many terms point to roles in chromatin organization and protein modification, suggesting that this module governs high‐level regulatory changes. Module 17 has much higher activity in the R genotype, and within R, it was more expressed under low N (Figure 5D). This pattern suggests that Module 17 may underpin the R genotype's ability to cope with N starvation—possibly by altering gene expression programs via chromatin remodeling or post‐translational modifications when N is scarce. Within this module, we again identified an uncharacterized transcript group, N_R___TRINITY_DN7789_c0_g2_i2.gen, showing a strong genotype‐by‐nitrogen dependent expression, making it a prime candidate for controlling NUE, as it may encode a regulatory factor that is activated in the efficient genotype under N stress.

#### 3.3.5 Module 24

Appears to be involved in cellular processes related to protein regulation, amino acid metabolism, and plant defense responses, including non‐photochemical quenching (NPQ) and energy dissipation in photosystems. These functions imply a role in fine‐tuning photosynthetic light capture and stress response (NPQ). Transcript groups in this module were predominantly activated in the R genotype under high N, with one notable exception: a gene encoding the chlorophyll a/b‐binding protein CP26 (a light‐harvesting complex component, SCA2_2__c95272f1p11035.gen) was down‐regulated in R (while upregulated in NR) (Figure 5E). The repression of a light‐harvesting protein in the R genotype under high N (despite overall higher chlorophyll content in R) might seem counterintuitive, but it could reflect a strategy to avoid over‐excitation of photosystems. CP26 proteins have a photoprotective role by binding xanthophylls to dissipate energy excess as heat (Dall'Osto et al. 2005). As R plants have more total chlorophyll and enhanced CO_2_ fixation capacity, reducing certain antenna proteins like CP26 could help balance light absorption with carbon metabolism, thus optimizing photosynthetic efficiency, since energy dissipation is not required. In contrast, the NR genotype upregulating CP26 might be trying to dissipate energy not being used by the impaired and less efficient photosystem (Bassi et al. 2018). The enrichment of defense and stress‐related terms indicates that the R genotype invests in photoprotection and stress mitigation mechanisms when N is abundant, thereby safeguarding its photosynthetic apparatus.

#### 3.3.6 Module 29

This module is highly activated only in the R genotype (Figure 5F). Enriched functions span various stress and signaling pathways, notably phosphorus and phosphate metabolism, response to external biotic stimuli, general stress responses, apoptosis, and oxylipin biosynthesis (lipid‐based signaling) and lipid oxidation. The fact that these processes are induced only in the R genotype suggests a unique preparedness or resilience inherent to the NUE‐efficient genotype. Oxylipins (like jasmonates) are signaling molecules in plant stress and development, so their biosynthesis being prominent in R could mean this genotype uses hormonal signals to orchestrate a robust response to its environment, consistent with findings in wheat where N status strongly influenced oxylipin‐mediated defense responses (Cascant‐Vilaplana et al. 2023).

#### 3.3.7 Modules 26, 37, 81, 93, and 105

It shows an association with RUBISCO, total chlorophyll, chlorophyll a, chlorophyll b, and/or PEPCASE levels (Figure 4 and Supplementary Figure 4). These modules are enriched in GO terms related to plant cell wall organization or biogenesis, negative regulation of growth, auxin polar transport, cellular component organization, cell wall polysaccharide metabolic process, hemicellulose metabolic process, peptide metabolic process, regulation of gene expression, and defense response (Supplementary Table 2). In contrast, modules correlated with PEPCASE (Figure 4) are associated with various combinations of metabolites and N conditions. No modules correlate with both genotype and N condition independently (Figure 4). As mentioned, one module (module 123) did correlate with the interaction between N condition and genotype. Module 123 is enriched in transcript groups involved in methylation and defense response. The absence of modules correlating N condition and genotype and the interaction between both, suggests genetic mechanisms underlying N use efficiency (NUE) differ from those governing the plant's response to N availability. Alternatively, the results may reflect more complex interactions, such as non‐monotonic relationships, that correlation analysis does not capture.

### 3.4 Transcription Associated Proteins (TAPs) and N Response

**FIGURE 6:**
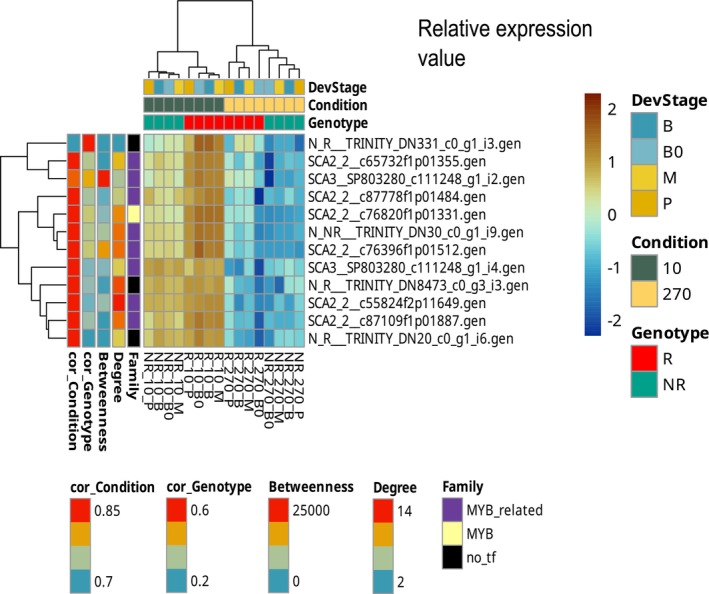
Transcription associated proteins (TAPs) in module 20. TAPs were identified by domain detection with HMMER v3.3.2 against Pfam v34 and classified into families using PlnTFDB. Expression profile of module 20, which is enriched in MYB/MYB‐related TAPs (75% of its transcript groups). Nodes with high degree and betweenness centrality are indicated, highlighting potential regulatory hubs. Relative expression values correspond to *z*‐score of variance‐stabilized counts from DESeq2. NR indicates non‐responsive genotype (NR: RB937570), R, responsive genotype (R: RB975375). Leaf segments/developmental stage = B0: Base 0, B: base, M: middle, P: tip, nitrogen condition = 10: 10 mg N kg^−1^ sand, 270: 270 mg N kg^−1^ sand.

TAPs often serve as master regulators of stress and developmental processes. We surveyed our DEGs for known TAP domains and identified 39 transcript groups belonging to TAP families. Strikingly, over half of these (57.4%) fell into the MYB/MYB‐related family, a proportion significantly higher than expected by chance (*p* < 0.01). Other represented families include bHLH (10.6%), CCAAT (8.5%), WRKY (6.38%), ARF and TRAF (4.25%), and a few others. Previous studies have linked MYB proteins to N use in grasses; for example, Gelli et al. (2014) found that several MYB genes were upregulated in nitrogen‐efficient sorghum genotypes, and Yang et al. (2019) reported MYB‐family regulators among the top differentially expressed genes in low‐N‐tolerant sugarcane varieties. Our data reinforce those findings, suggesting that MYB transcription factors are central players in the regulatory network underlying NUE. The 39 TAPs exhibited diverse expression patterns: some responded primarily to N level, others to genotype differences, and a few to both (Figure S2). This indicates that the regulatory control of NUE is distributed across several layers, that is, certain TAPs act as general N‐responsive switches, while others are tied to the genetic background. Notably, module 20 is highly enriched with TAPs (75% of transcript groups present are TAPs, predominantly from MYB or MYB‐related families), and they respond to genotype. These factors were activated in low N conditions in both genotypes, with a stronger activation in the R genotype. We identified several hub transcript groups (nodes with high (top 10%) connectivity (degree) and centrality (betweenness)) in this module that likely act as key regulators (Figure 6). For instance, SCA3__SP803280_c108399_g1_i1 annotated with roles in protein ubiquitination and response to salt stress, and SCA3__SP803280_c111248_g1_i2 annotated with *cis*‐regulatory DNA binding, cellular response to potassium ion, potassium ion transport regulation and response to chitin. The presence of these functions suggests that the hub TAPs in this module may integrate nutrient signals (N and K) with defense and stress signals. Potassium is often a critical co‐factor in maintaining high photosynthetic rates, and it is functionally linked to N use. For example, K^+^ serves as a counter‐ion for nitrate (NO_3_^−^), balancing its uptake and facilitating its transport, and optimizes CO_2_ conductance and PNUE (Xu et al. 2020; Cao et al. 2025). Additionally, the function “response to chitin” for a hub gene indicates overlap between nutrient stress responses and pathogen defense pathways, consistent with models of integrated biotic–abiotic stress signaling, as we already mentioned for chitinases (Saijo and Loo 2020). The high degree and central betweenness of these transcript groups mean that they connect to many other transcript groups and lie on many shortest paths in the network, respectively. This central position in the network, together with their differential activation under low N, suggests that Module 20 orchestrates a coordinated low‐N response across the plant.

Other functions annotated in Module 20 are related to floral development, reproductive processes, and senescence regulation, suggesting that prolonged N stress might push plants to alter their developmental program, for instance, entering senescence under nutrient‐poor conditions. Such responses are well documented in plants, with N deficiency often causing earlier flowering and remobilization of nutrients from leaves (senescence) as a survival strategy (Havé et al. 2017; Sanagi et al. 2021; Sakuraba 2022; Fan et al. 2023).

Taken together, our transcriptomic and network analyses provide a multilayered view of how sugarcane responds to and manages N. The N‐responsive genotype (R) deploys a complex but coordinated strategy to enhance its PNUE. Under ample N, it upregulates carbon assimilation pathways (e.g., glycolysis and PEPCase gene), and numerous regulatory genes, including many MYB transcription factors, to fine‐tune metabolism and mitigate stress, particularly in older leaf segments. These adjustments allow the R genotype to maintain higher CO_2_ fixation than the less efficient genotype. By contrast, the non‐responsive genotype (NR) relies more on basal responses such as broadly boosting photosynthesis‐related transcript groups and activating general stress signals. While those responses may provide a short‐term benefit, they do not translate into sustained performance, especially under prolonged or varying N conditions.

Our co‐expression network further pinpoints key modules and hub genes that differentiate the R and NR responses. Several network modules uniquely induced in the R genotype, notable modules 17, 24 and 29, and some nodes in module 15 harbor promising candidate genes that could underlie the genetic basis of superior NUE in sugarcane. These include known functional players like PEPCase, as well as many uncharacterized transcript groups. Additionally, we identified module 20 as a conserved N‐responsive module (active in both genotypes) under low N and enriched for MYB transcription factors and other regulators; this module likely represents a core stress‐adaptive program activated under N limitation in sugarcane. From an applied perspective, these findings forge valuable links between molecular data and whole‐plant performance traits that can be exploited in sugarcane improvement towards varieties with enhanced NUE and PNUE. Future research should expand on a larger set of genotypes with a more nuanced N availability regime, in order to improve our understanding of the full spectrum of genetic or dose‐dependent variation.

## Author Contributions

Conceptualization: M.M. (lead experimental part), L.M. (lead experimental part), D.M.R.‐P. (lead computational part). Data curation: J.M.M.‐P., D.M.R.‐P. Formal analysis: J.M.M.‐P. (lead), D.M.R.‐P. (supporting). Funding acquisition: D.M.R.‐P., M.M. Investigation: L.M., D.B. Methodology: J.M.M.‐P., D.M.R.‐P. Project administration: D.M.R.‐P. (lead), L.M. (equal). Resources: M.M., D.M.R.‐P., L.M. Supervision: D.M.R.‐P. Validation: J.M.M.‐P. Visualization: J.M.M.‐P. Writing – original draft: J.M.M.‐P. (lead), D.M.R.‐P. (supporting). Writing – review and editing: J.M.M.‐P. (lead), D.M.R.‐P. (lead), L.M. (supporting), M.M. (critical review), D.B. (commentary).

## Supporting information


**Data S1:** Supporting Information.

## Data Availability

Raw RNA‐Seq datasets were submitted to the Short Read Archive maintained by NCBI, under the BioProject number PRJNA1176579. All computer code used for analyses is available here: https://github.com/labbces/NRGSC Further data was deposited in FigShare (https://figshare.com/projects/Nitrogen_‐_Gene_co‐expression_networks_highlight_key_nodes_associated_with_ammonium_nitrate_response_in_sugarcane/242225): *processed expression matrices* (DESeq2‐normalized counts), a *de novo* transcriptome assembly (3.8 million transcripts clustered into 2.48 million transcript groups), co‐expression network (199 modules with 1109 nodes) and complete tables for GO functional enrichment.
