# Supplementary material for: Gene Co‐Expression Networks Highlight Key Nodes Associated With Ammonium Nitrate in Sugarcane
Source: Physiol Plant. 2025 Oct 31;177(6):e70612. doi: 10.1111/ppl.70612 (PMC12576763; doi:10.1111/ppl.70612)
Supplement: Supplementary file 1 — Data S1: Supporting Information. [file PPL-177-e70612-s001.pdf]

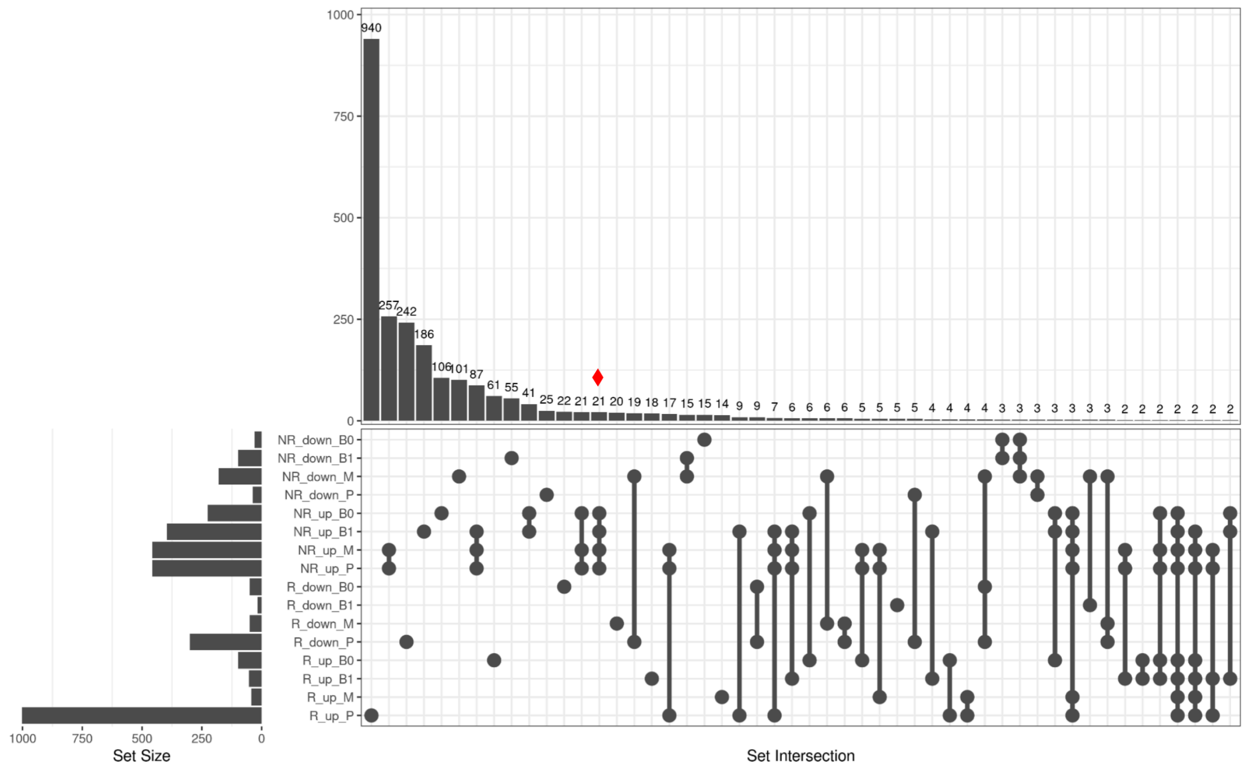

Supplementary Figure 1. **All Sets of differentially expressed transcript groups; shared and unique across the different conditions.** NR indicates Non-responsive genotype, R, responsive genotype, Up marks related to the transcript groups overexpressed in high nitrogen availability. Down points related to transcript groups overexpressed in low nitrogen availability. B0, B1, M and P differentiate Base 0, Base, Medium and Tip leaf segments. Red diamond indicate a set of transcript groups always up-regulated in NR genotype in high nitrogen availability irrespective of the leaf segment.

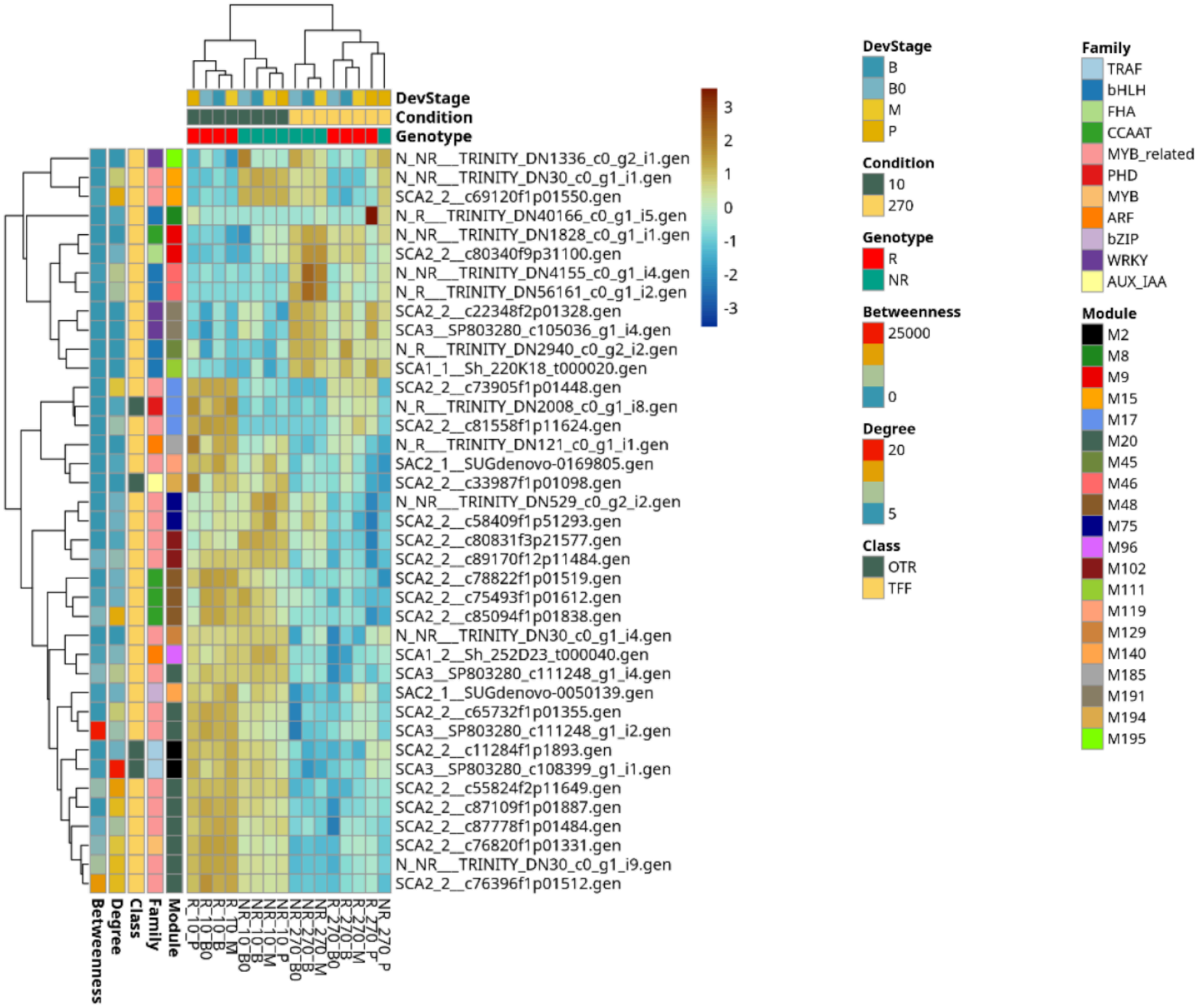

Supplementary Figure 2. **Expression patterns of transcription associated proteins (TAPs) in all the network**. TAPs were identified by domain detection with HMMER v3.3.2 against Pfam v34 and classified into families using PlnTFDB. Relative expression values correspond to z-score of variance-stabilized counts from DESeq2.

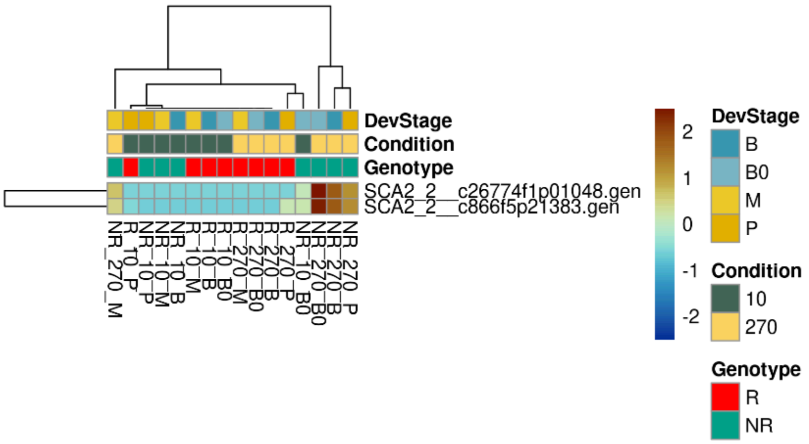

Supplementary Figure 3. **Expression pattern of module 123 correlated to the interaction of condition and genotype spearman coefficient > abs|0.7|**. Enriched in transcript groups involved in methylation and defense response. Relative expression values correspond to z-score of variance-stabilized counts from DESeq2. The modules were generated using the Markov Cluster Algorithm (mcl), with an inflation value of 1.8 on the correlation matrix.

Supplementary Table 1. GO functional enrichment for module 123. p-values were calculated using the TopGO R package, multiple testing were corrected using Benjamini-Hochberg method.

GO.ID	Term	Annotated	Significant	Expected	Classic	p.adj
GO:0032259	methylation	33	2	0.09	0.0019	0.0038
GO:0006952	defense response	151	2	0.41	0.0409	0.0409

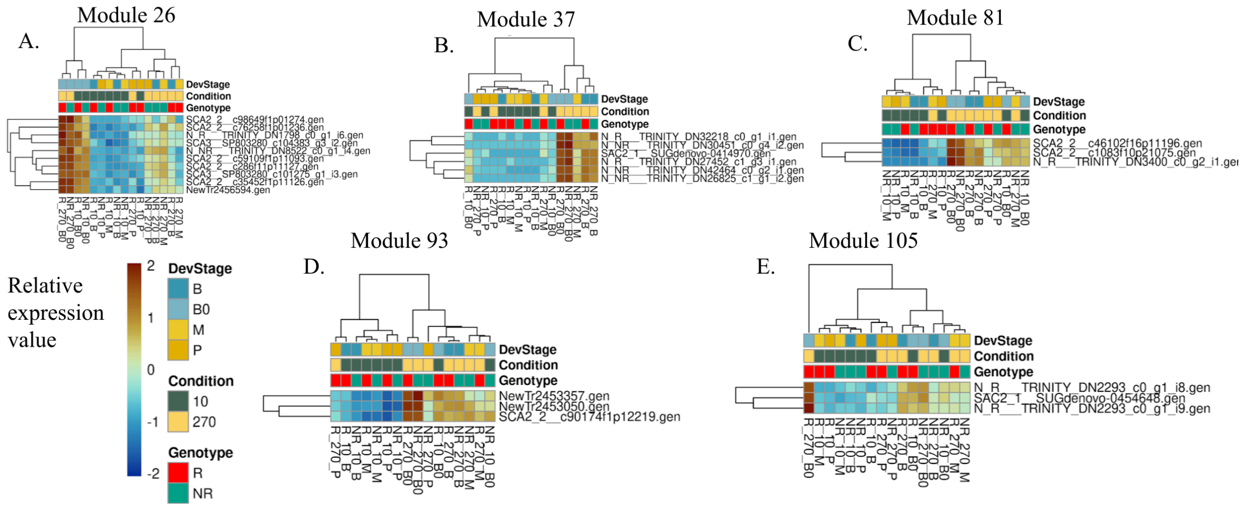

Supplementary Figure 4. **Expression pattern of modules 26, 37, 81, 93, 105 123 correlated with RUBISCO, total chlorophyll, chlorophyll a, chlorophyll b, and/or PEPCASE**. Spearman coefficient > |0.7|. Relative expression values

correspond to z-score of variance-stabilized counts from DESeq2. The modules were generated using the Markov Cluster Algorithm (mcl), with an inflation value of 1.8 on the correlation matrix.

Supplementary Table 2. GO functional enrichment for modules 26, 37, 81, 93, 105 123. p-values were calculated using the TopGO R package, multiple testing were corrected using Benjamini-Hochberg method.

GO.ID	Term	Annotated	Significant	Expected	Classic	p.adj	module
GO:0071669	plant-type cell wall organization or bio...	10	3	0.13	0.0002	0.002	26
GO:0009926	auxin polar transport	1	1	0.01	0.0134	0.0149	26
GO:0035265	organ growth	1	1	0.01	0.0134	0.0149	26
GO:0045926	negative regulation of growth	1	1	0.01	0.0134	0.0149	26
GO:0046620	regulation of organ growth	1	1	0.01	0.0134	0.0149	26
GO:0046621	negative regulation of organ growth	1	1	0.01	0.0134	0.0149	26
GO:0048638	regulation of developmental growth	1	1	0.01	0.0134	0.0149	26
GO:0048640	negative regulation of developmental gro...	1	1	0.01	0.0134	0.0149	26
GO:2000012	regulation of auxin polar transport	1	1	0.01	0.0134	0.0149	26
GO:0048519	negative regulation of biological proces...	20	2	0.27	0.0271	0.0271	26
GO:0016043	cellular component organization	57	3	0.23	0.00043	0.0005	81
GO:0071840	cellular component organization or bioge...	60	3	0.24	0.0005	0.0005	81
GO:0010383	cell wall polysaccharide metabolic proce...	1	1	0	0.0013	0.0033	93
GO:0010410	hemicellulose metabolic process	1	1	0	0.0013	0.0033	93
GO:0010411	xyloglucan metabolic process	1	1	0	0.0013	0.0033	93
GO:0044347	cell wall polysaccharide catabolic proce...	1	1	0	0.0013	0.0033	93

GO:0085030	symbiotic process benefiting host	1	1	0	0.0013	0.0033	93
GO:2000895	hemicellulose catabolic process	1	1	0	0.0013	0.0033	93
GO:2000899	xyloglucan catabolic process	1	1	0	0.0013	0.0033	93
GO:0016998	cell wall macromolecule catabolic proces...	11	1	0.01	0.0148	0.0296	93
GO:0044036	cell wall macromolecule metabolic proces...	11	1	0.01	0.0148	0.0296	93
GO:0009251	glucan catabolic process	13	1	0.02	0.0174	0.0313	93
GO:0006073	cellular glucan metabolic process	19	1	0.03	0.0255	0.0328	93
GO:0044042	glucan metabolic process	19	1	0.03	0.0255	0.0328	93
GO:0044264	cellular polysaccharide metabolic proces...	19	1	0.03	0.0255	0.0328	93
GO:0044403	symbiotic process	19	1	0.03	0.0255	0.0328	93
GO:0044262	cellular carbohydrate metabolic process	21	1	0.03	0.0282	0.0338	93
GO:0071554	cell wall organization or biogenesis	33	1	0.04	0.0443	0.0483	93
GO:0000272	polysaccharide catabolic process	34	1	0.05	0.0456	0.0483	93
GO:0016052	carbohydrate catabolic process	36	1	0.05	0.0483	0.0483	93
GO:0006518	peptide metabolic process	38	3	0.15	0.00012	0.0026	105
GO:0043603	cellular amide metabolic process	49	3	0.2	0.00027	0.0026	105
GO:0010556	regulation of macromolecule biosynthetic...	69	3	0.28	0.00076	0.0026	105
GO:2000112	regulation of cellular macromolecule bio...	69	3	0.28	0.00076	0.0026	105
GO:0009889	regulation of biosynthetic process	70	3	0.28	0.0008	0.0026	105

GO:0031326	regulation of cellular biosynthetic proc...	70	3	0.28	0.0008	0.0026	105
GO:0051171	regulation of nitrogen compound metaboli...	70	3	0.28	0.0008	0.0026	105
GO:0010468	regulation of gene expression	71	3	0.29	0.00083	0.0026	105
GO:0080090	regulation of primary metabolic process	71	3	0.29	0.00083	0.0026	105
GO:0060255	regulation of macromolecule metabolic pr...	72	3	0.29	0.00087	0.0026	105
GO:0031323	regulation of cellular metabolic process	76	3	0.31	0.00102	0.0026	105
GO:0019222	regulation of metabolic process	78	3	0.31	0.00111	0.0026	105
GO:1901566	organonitrogen compound biosynthetic pro...	78	3	0.31	0.00111	0.0026	105
GO:0009059	macromolecule biosynthetic process	92	3	0.37	0.00183	0.0034	105
GO:0010467	gene expression	92	3	0.37	0.00183	0.0034	105
GO:0034645	cellular macromolecule biosynthetic proc...	92	3	0.37	0.00183	0.0034	105
GO:0044267	cellular protein metabolic process	121	3	0.49	0.0042	0.0074	105
GO:0050794	regulation of cellular process	129	3	0.52	0.00509	0.0084	105
GO:0044271	cellular nitrogen compound biosynthetic ...	131	3	0.53	0.00533	0.0084	105
GO:0050789	regulation of biological process	150	3	0.6	0.00803	0.0117	105
GO:0006952	defense response	151	3	0.61	0.00819	0.0117	105
GO:0019538	protein metabolic process	155	3	0.62	0.00887	0.0121	105
GO:0065007	biological regulation	188	3	0.76	0.01588	0.0205	105
GO:0034641	cellular nitrogen compound metabolic pro...	190	3	0.77	0.01639	0.0205	105
GO:0044249	cellular biosynthetic process	215	3	0.87	0.0238	0.0286	105

GO:0006950	response to stress	224	3	0.9	0.02693	0.0311	105
GO:0044260	cellular macromolecule metabolic process	233	3	0.94	0.03032	0.0325	105
GO:1901576	organic substance biosynthetic process	233	3	0.94	0.03032	0.0325	105
GO:0009058	biosynthetic process	248	3	1	0.03659	0.0379	105
GO:1901564	organonitrogen compound metabolic proces...	265	3	1.07	0.04468	0.0447	105
